# Interleukin-1 Receptors Are Differentially Expressed in Normal and Psoriatic T Cells

**DOI:** 10.1155/2014/472625

**Published:** 2014-02-10

**Authors:** Attila Bebes, Ferenc Kovács-Sólyom, Judit Prihoda, Róbert Kui, Lajos Kemény, Rolland Gyulai

**Affiliations:** ^1^Department of Dermatology and Allergology, University of Szeged, Korányi fasor 6, Szeged 6720, Hungary; ^2^Dermatological Research Group of the Hungarian Academy of Sciences, University of Szeged, Korányi fasor 6, Szeged 6720, Hungary; ^3^Department of Dermatology, Venereology and Oncodermatology, University of Pécs, Kodály Z. u. 20, Pécs 7624, Hungary

## Abstract

This study was carried out to examine the possible role of interleukin-1 (IL-1) in the functional insufficiency of regulatory T cells in psoriasis, by comparing the expression of IL-1 receptors on healthy control and psoriatic T cells. Patients with moderate-to-severe chronic plaque psoriasis and healthy volunteers, matched in age and sex, were selected for all experiments. CD4^+^CD25^−^ effector and CD4^+^CD25^+^CD127^low^ regulatory T cells were separated and used for the experiments. Expression of the mRNA of IL-1 receptors (IL-1R1, IL-1R2, and sIL-1R2) was determined by quantitative real-time RT-PCR. Cell surface IL-1 receptor expression was assessed by flow cytometry. Relative expression of the signal transmitting IL-1 receptor type 1 (IL-1R1) mRNA is higher in resting psoriatic effector and regulatory T cells, and activation induces higher IL-1R1 protein expression in psoriatic T cells than in healthy cells. Psoriatic regulatory and effector T cells express increased mRNA levels of the decoy IL-1 receptors (IL-1R2 and sIL-1R2) upon activation compared to healthy counterparts. Psoriatic T cells release slightly more sIL-1R2 into their surrounding than healthy T cells. In conclusion, changes in the expression of IL-1 receptors in psoriatic regulatory and effector T cells could contribute to the pathogenesis of psoriasis.

## 1. Introduction

Psoriasis, a common inflammatory skin disorder affecting 1-2% of individuals in Western societies, is caused by genetic predisposition and can be triggered or affected by various environmental provoking factors, such as mechanical stress (Koebner phenomenon), infections, emotional stress, diet, body mass index, alcohol consumption, smoking, certain drugs, and climatic effects [1–3]. Psoriatic skin lesions are infiltrated with activated T cells and hyperstimulatory antigen presenting cells [4–6]. Recently published studies suggest that intralesional activated T cells produce cytokines that trigger primed basal stem cell keratinocytes to proliferate and perpetuate skin inflammation. The interaction between keratinocytes and immune cells via autocrine and paracrine network of cytokines is a key component in the development of psoriasis [7, 8].

Interleukin-1 (IL-1) is a potent inflammatory cytokine implicated in host-defence responses to injury and infection. Several factors (IL-1 receptors, agonists, and antagonists) are involved in the regulation of IL-1 activity [9]. The type 1 receptor (IL-1R1) is described as a signal transmitting receptor, triggered by both IL-1*α* and IL-1*β* ligands. The intracellular domain of IL-1R1 is responsible for initialising the inflammatory signalling processes in target cells. The type 2 IL-1 receptors (IL-1R2) are decoy receptors, as they are lacking the intracellular signal transmitting domain for mediating the IL-1 effect. IL-1R2 can be found associated with the plasma membrane and in soluble, secreted forms. Both of these receptor forms strongly bind IL-1; however, they are unable to initialise the IL-1 signalling pathway. Soluble IL-1R2 protein is produced by shedding from the cell surface or synthesised in a soluble form from a distinct gene (sIL-1R2). The contribution of IL-1 and related signalling to inflammatory skin diseases and to psoriasis pathogenesis is supported by several studies [10, 11].

According to our recently published data, psoriatic CD4^+^CD25^+^ regulatory T cells (Treg) are functionally defective in suppressing activated CD4^+^CD25^−^ effector T cell (Teff) proliferation compared to healthy Treg cells [12]. However, the reasons for regulatory T cell deficiency remain mostly unknown. Since IL-1 signalling leads to the release of several proinflammatory cytokines, including TNF*α*, IL-17A [13], and IL-6, it has been implicated in preventing immune suppression by regulatory T cells [14]. Therefore, we hypothesised that the IL-1 signalling pathway may be involved in the functional deficiency of regulatory T cells in psoriasis. This study aims to compare the expression of IL-1 receptor isoforms in Treg and Teff cells from psoriatic and healthy individuals.

## 2. Materials and Methods

### 2.1. Patients

Patients with moderate-to-severe chronic plaque type psoriasis and healthy volunteers, matched in age and sex, were selected for all experiments. Psoriatic patients were either untreated or had only received topical therapy during the last 4 weeks before sampling. Samples were collected from at least four patients and four healthy volunteers for each experiment. The study was approved by the Human Investigation Review Board of the University of Szeged, compiling with the ethical standards of research and in accordance with the Helsinki Declaration. Written informed consent was obtained from all donors involved in the study.

### 2.2. Reagents

Human T Regulatory Lymphocyte Isolation Set, anti-CD127-PE, anti-CD45RO-FITC, anti-CD25-APC, PE and APC conjugated streptavidin, and human recombinant IL-2 protein were purchased from BD Biosciences (San Jose, CA, USA). All flow cytometry and flow sorting experiments were done on FACSCalibur flow cytometer and data were analysed with CellQuest software (BD Biosciences). RPMI-1640 medium, anti-CD3/CD28 coated bead, and TRIzol reagent were from Life Technologies (Carlsbad, CA, USA). Foetal bovine serum (FBS) was obtained from HyClone Laboratories, Inc. (South Logan, Utah, USA). Anti-CD4-PerCP, biotinylated anti-IL-1R1, anti-IL-1R2, and Human sIL-1R2 Quantikine ELISA Kit were from R&D Systems (Minneapolis, MN, USA), and anti-GARP-PE (LRRC32) was from ENZO Life Sciences (Farmingdale, NY, USA). Antibiotic/Antimycotic Solution, L-glutamine, MEM's Vitamin Solution, and sodium-azide were purchased from Sigma-Aldrich (Saint Louis, MO, USA). Ficoll Paque was acquired from GE Healthcare Biosciences (Uppsala, Sweden).

### 2.3. Isolation and Activation of Regulatory and Effector T Cells

After gradient centrifugation of peripheral blood from psoriatic patients and healthy volunteers using Ficoll Paque, CD4^+^ cells were separated from peripheral blood mononuclear cells by negative selection using antibody-coupled magnetic beads. Activation of the cells was carried out by incubation with CD3/CD28 beads (1 : 4 bead to cell ratio) for 1, 6, and 24 hours (RT-PCR experiments), 48 hours (for flow cytometry), and 72 hours (ELISA experiments), following the instructions of the manufacturer.

Different T cell subpopulations were identified by flow cytometer using CD45RO and CD25 labelling. CD45RO and CD25 double negative cells were considered as naïve T cells (TN), CD45RO negative and CD25 positive cells as naïve regulatory T cells (TNreg), CD45RO positive and CD25 negative cells as memory T cells (TM), and CD45RO and CD25 double positive cells as regulatory T cells (Treg); in the case of activated cells anti-GARP antibody was used instead of anti-CD25 for discriminating CD4^+^ subpopulations, as previously described [15].

CD4^+^ and CD4^+^CD25^+^ cells were separated with Human T Regulatory Lymphocyte Isolation Set for real-time RT-PCR and ELISA experiments. CD127^low^ cells were further selected from the CD4^+^CD25^+^ population by anti-CD127 antibodies and flow cytometer assisted sorting as described previously [16].

### 2.4. Cell Culture

T cells were maintained in RPMI-1640 medium supplemented with 10% FBS, 1% Antibiotic/Antimycotic Solution, 1% L-glutamine, and MEM's Vitamin Solution at 37°C in a humidified atmosphere containing 5% CO_2_.

### 2.5. Real-Time RT-PCR

Regulatory and effector T cells were activated at indicated times and total RNA was isolated using TRIzol reagent; RNA concentration was determined by A260 values. cDNA was synthesised from 1 *μ*g of total RNA using the Bio-Rad iScript cDNA Synthesis Kit, and RT-PCR experiments were done on the iCycler IQ Real-Time PCR machine of Bio-Rad (Hercules, CA, USA). The abundance of each gene of interest was normalised to the expression of 18S ribosomal rRNA gene from each examined sample; data are expressed as an arbitrary number proportional to the mRNA level.

### 2.6. Flow Cytometry

CD4^+^ T cells were cultured overnight after the separating procedure. Activated cells were incubated for two days with CD3/CD28 coated beads in the presence of 10 U/mL IL-2. Control cells were incubated without beads and IL-2. After two days cells were harvested and washed once with PBS. CD4^+^ cells were >95% pure as verified by anti-human CD4-PerCP labelling. Different cell populations were identified by staining with anti-human CD45RO-FITC and anti-human CD25-APC or anti-human GARP-PE 45 minutes on ice. Cells were labelled with biotinylated anti-human IL-1R1 or biotinylated anti-human IL-1R2 monoclonal antibodies or appropriate isotype controls in 1 *μ*g/mL concentration for 45 minutes on ice. After washing two times with FACS buffer (1% FBS + 0.1% sodium-azide in PBS) PE- or APC-conjugated streptavidin was added and incubated for 30 minutes on ice. Samples were washed and resuspended in 500 *μ*L FACS buffer; staining was measured using FACSCalibur.

### 2.7. Determination of Secreted sIL-1R2 by Using ELISA Technique

Hundred-thousand normal and psoriatic effector and regulatory T cells were grown per wells in a ninety-six well plate. Supernatants of control and CD3/CD28 activated cells were harvested after three days of incubation. Soluble IL-1R2 protein level in the supernatants was determined by using an ELISA Kit for sIL-1R2 following the instructions of the manufacturer.

### 2.8. Statistical Analysis

All data were statistically analysed and compared for significance using one-way ANOVA (Holm-Sidak method) for multiple comparisons in SigmaPlot software (Systat Software, Inc., Chicago, IL, USA).

## 3. Results

### 3.1. Psoriatic Naïve, Memory, Naïve Treg, and Treg Cell Populations Are Quantitatively Identical to Healthy Cell Populations

**Figure 1:**
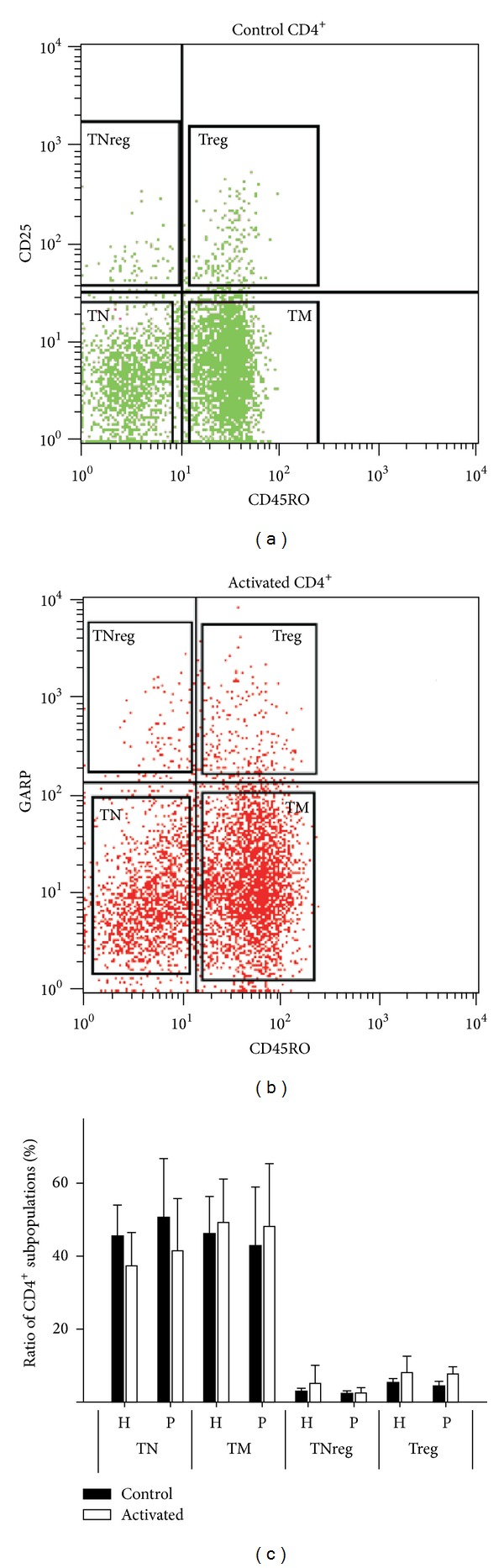
Distribution of CD4^+^ T lymphocyte subpopulations is not different in healthy and psoriatic peripheral blood samples. CD4^+^ T cells were isolated from healthy and psoriatic donors and were activated for two days with CD3/CD28 beads in presence of IL-2; control cells were incubated without any treatment. Cells were stained with anti-CD45RO-FITC and anti-CD25-APC to identify T cell subpopulations such as naïve T (TN, CD45RO^−^CD25^−^), memory T (TM, CD45RO^+^CD25^−^), naïve regulatory T (TNreg, CD45RO^−^CD25^+^), and regulatory T cells (Treg, CD45RO^+^CD25^+^) (a). Anti-GARP-PE antibody was used instead of anti-CD25 to differentiate activated cells (b). Representative scatterplots from healthy samples are shown. Percentage of cells in each population was determined and shown as the mean of four independent samples from healthy (H) and psoriatic (P) peripheral blood (c). Control resting cells and activated cells are represented by black and white bars, respectively.

CD4^+^ T cells were isolated from PBMC fractions of healthy and psoriatic peripheral blood samples using magnetic bead technique. Naïve (TN), memory (TM), naïve regulatory (TNreg), and regulatory T cell (Treg) subpopulations were identified by CD45RO and CD25/GARP labelling (Figures 1(a) and 1(b)). Similar distribution of T cell populations was found in resting normal and psoriatic samples and there was no significant difference in the cell number of control and psoriatic T cell subgroups. About 40% naïve, 45% memory, and 2–5% TNreg and Treg were detected among CD4^+^ cells (Figure 1(c)) and no significant changes were observed two days after CD3/CD28 stimulation.

### 3.2. Psoriatic Treg Cells Display a Consistently Increased IL-1R1 Gene Expression Compared to Healthy Counterparts

**Figure 2:**
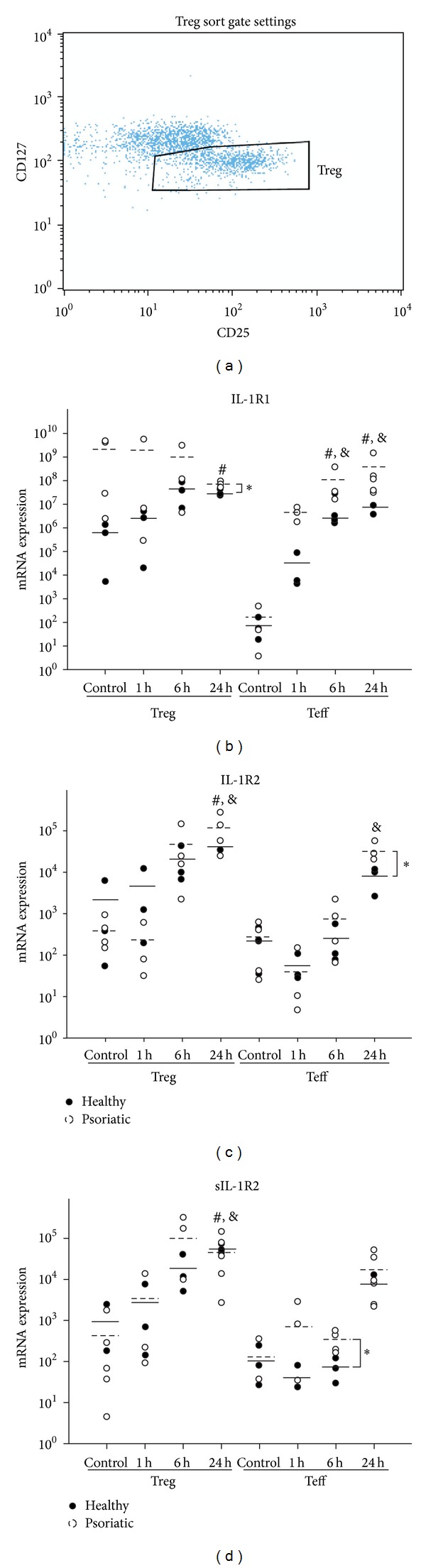
IL-1 receptor mRNA expression is different in psoriatic T cells compared to healthy counterparts. Healthy and psoriatic effector (Teff) and regulatory (Treg) T cells were separated from PBMC fractions. Teff cells were isolated via negative selection method by magnetic beads. Treg cells were sorted from CD4^+^CD25^+^ population using anti-CD127 labelling; sorting gate was set for CD25^high^CD127^low^ cells (a). Isolated Treg and Teff cells were activated with anti-CD3/CD28 coated beads for indicated times and mRNA expression changes of IL-1 receptor isoforms were determined by real-time RT-PCR at 0 h baseline control and 1 h, 6 h, and 24 h after CD3/CD28 stimulation. Comparison of the expression of IL-1R1 (b), IL-1R2 (c), and sIL-1R2 (d) genes in T cells from healthy (*n* = 3, black circles) and psoriatic samples (*n* = 4, white circles); mean values are indicated by solid and dashed lines, respectively. Gene expression values are represented as arbitrary numbers normalised to the expression of 18S rRNA gene. *—significant difference (*P* < 0.05) between healthy and psoriatic samples and significant difference (*P* < 0.05) between baseline and activated mRNA levels of healthy (#) and psoriatic (&) samples.

In order to investigate the IL-1R1 mRNA expression in CD4^+^ T cell subpopulations, peripheral blood mononuclear cells were separated into CD4^+^CD25^−^ effector cells (Teff) and CD4^+^CD25^+^CD127^−^ regulatory cells (Treg) using a combination of magnetic bead and flow cytometer assisted sorting method (Figure 2(a)). Total RNA was isolated from control and CD3/CD28 bead activated cells, and IL-1R1 mRNA level was detected by real-time RT-PCR (Figure 2(b)). Control resting Teff cells expressed very low levels of IL-1R1 mRNA both in healthy and psoriatic samples. Activation significantly induced IL-1R1 gene expression in Teff cells; the induction was more pronounced in psoriatic Teff cells at all time points examined, although the differences between healthy and psoriatic samples were not statistically significant.

Resting Treg cells showed higher IL-1R1 gene expression compared to Teff cells, with the psoriatic Treg cells showing elevated mRNA levels over healthy counterparts. IL-1R1 mRNA expression was consistently induced in healthy Treg cells reaching the highest level at 6 hours and remaining elevated at 24 hours after CD3/CD28 stimulation (statistical significance at 24 hours, Figure 2(b)). Psoriatic T cells, however, did not respond with further increase of IL-1R1 mRNA levels to T cell receptor activation signals; even a slight decrease was observed at 24 hours. IL-1R1 gene expression was still higher in activated psoriatic Treg cells compared to healthy counterparts (statistical significance at 24 hours, Figure 2(b)).

### 3.3. The Induction of IL-1R2 and sIL-1R2 Gene Expressions following Cell Activation Is More Prominent in Psoriatic Teff Cells Compared to Healthy Ones

The mRNA expression pattern of the two decoy IL-1 receptors (IL-1R2 and sIL-1R2) was strikingly similar. There was no significant difference in the baseline mRNA expression of the IL-1R2 (Figure 2(c)) and sIL-1R2 (Figure 2(d)) genes between healthy and psoriatic Teff or Treg cells. Upon CD3/CD28 activation, the mRNA expression of the decoy IL-1 receptors increased in every Teff and Treg samples examined (statistically significant differences at 24 hours after activation in psoriatic Teff IL-1R2 mRNA expression; and in both healthy and psoriatic Treg IL-1R2 and sIL-1R2 mRNA expression compared to baseline resting cells). After T cell receptor stimulation psoriatic T cells expressed higher levels of the decoy IL-1 receptors than healthy counterparts (significant differences between psoriatic and healthy Teff cells in IL-1R2 and sIL-1R2 mRNA expressions at 24 and 6 hours after activation, resp.).

### 3.4. Higher Percentage of Activated Psoriatic Treg Cells Expresses IL-1R1 Protein with Decreased Intensity Compared to Healthy Cells

**Figure 3:**
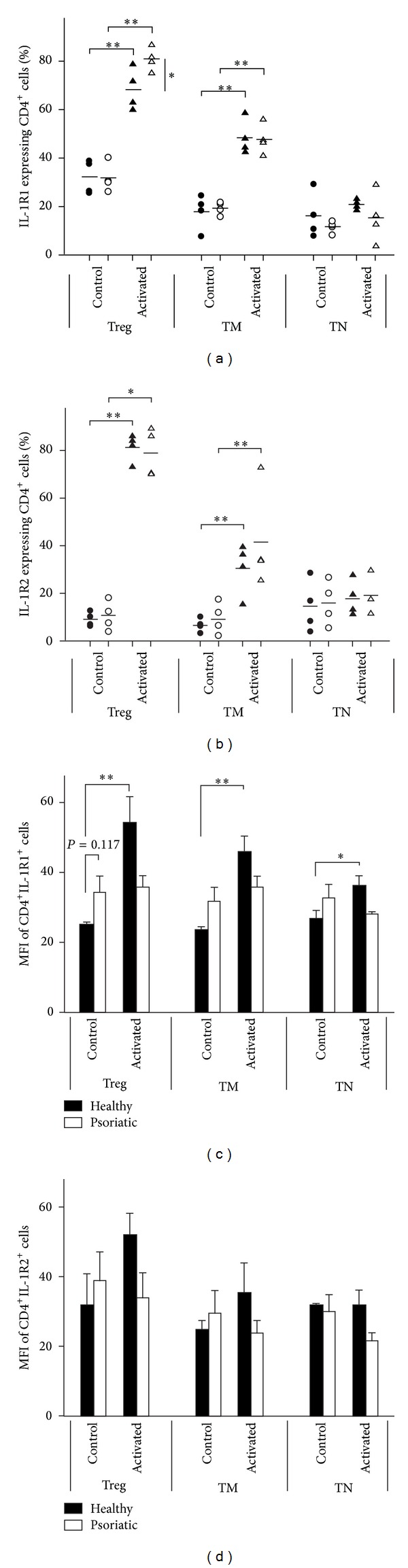
Expression of IL-1R1 and IL-1R2 proteins in healthy control and psoriatic CD4^+^ cells. Healthy and psoriatic CD4^+^ cells were isolated and labelled as described. Cell surface IL-1R1 (a) and IL-1R2 (b) proteins were stained with biotinylated primary antibodies and PE/APC-conjugated streptavidin. Percentage of positive cells of CD4^+^ Treg, TM, and TN subpopulations from individual samples is depicted (*n* = 4). Circles represent resting cells (CON), whereas CD3/CD28 stimulated cells (ACT) are indicated as triangles. Mean fluorescence intensities (MFI) of IL-1R1 (c) and IL-1R2 (d) positive cells are shown. *P < 0.05, **P < 0.01.

Normal and psoriatic CD4^+^ cells were cultured for two days in the presence (activated cells) or absence (control cells) of anti-CD3/CD28 coated beads and IL-2 (10 U/mL). Cell surface IL1R1 and IL-1R2 expressions were determined using flow cytometry. T cell subpopulations (TN, TM, TNreg, and Treg) were gated as previously described (Figure 1(a)). Percentage of cells showing IL-1R1 positivity was compared between control healthy and psoriatic samples (Figure 3(a)). In resting T cells, IL-1R1 is expressed at the highest percentage in the Treg subpopulation both in healthy and psoriatic samples, reaching 32.31% and 31.87%, respectively. Cell surface presence of IL-1R1 was notably lower in TM (17.89%/19.52%) and TN (16.21%/11.73%) subpopulations. The intensity of IL-1R1 receptor expression (Figure 3(c)) was higher in psoriatic Treg cells (mean fluorescent intensity, MFI = 34.26) compared to healthy counterparts (MFI = 24.84); however this difference was not significant (*P* = 0.117).

Upon activation, the number of IL-1R1 expressing cells displayed a vast increase, with a more pronounced rise in psoriatic Treg cells, resulting in a significant difference between the number of IL-1R1 positive cells in the healthy (68.4%) and psoriatic (80.8%) samples (Figure 3(a)). We detected a notable increase in IL-1R1 expression intensity (Figure 3(c)) two days after CD3/CD28 stimulation in healthy Treg cells (MFI = 54.11); this was not observed in psoriatic samples (MFI = 35.7).

The ratio of the IL-1R1 positive cells in the TM subpopulation increased to 48.39% in healthy and 47.8% in psoriatic samples two days after activation (Figure 3(a)). CD3/CD28 stimulated healthy TM cells presented a significant increase in the intensity of IL-1R1 staining (Figure 3(c), control MFI = 23.32, activated MFI = 45.83), whereas psoriatic TM cells displayed no change compared to resting cells (control MFI = 31.71, activated MFI = 35.86). No significant changes were detected in the number of IL-1R1 expressing TN cells following activation; however the expression intensity of the protein was increased in healthy TN as well. Taken together, these results demonstrate that activation induces IL-1R1 receptor expression on the surface of both healthy and psoriatic Treg and TM cells; however, receptor density is only increasing in healthy T cells upon CD3/CD28 activation.

### 3.5. IL-1R2 Expressing Treg and TM Cells Are Greatly Increased upon Activation

CD4^+^ cells were treated and labelled as previously described for flow cytometry analysis of healthy and psoriatic samples (Figure 1(a)). The T cell subpopulations were defined and IL-1R2 expression was determined. Most of the resting cells from all CD4^+^ subpopulations studied were negative for the IL-1R2 decoy receptor (Figure 3(b)). Unlike the IL-1R1 protein, the membrane bound decoy receptor was not expressed in a higher percentage among Treg cells. However, two days after activation most Treg cells displayed IL-1R2 positivity. Only a moderate increase was observed in IL-1R2 expressing TM and almost no change in TN subpopulations. Intensity of cell surface IL-1R2 expression (Figure 3(d)) was increased in healthy Treg and TM cells upon CD3/CD28 stimulation, whereas in the case of psoriatic samples we noticed a minor decrease in IL-1R2 expression intensity; these changes were not statistically significant.

### 3.6. Slightly Elevated sIL-1R2 Production in Psoriatic T Cells Compared to Healthy T Cells

**Figure 4:**
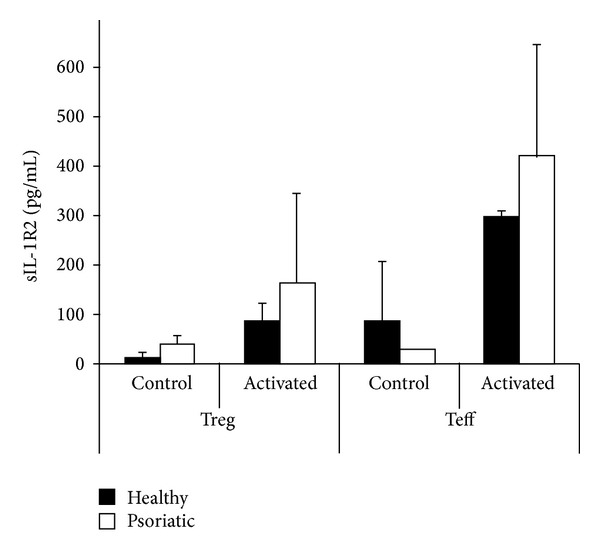
Soluble IL-1R2 protein secretion is not significantly different in healthy and psoriatic Treg and Teff cells. Cell populations were separated by magnetic bead and flow cytometer mediated cell sorting method as described. Supernatants of control and activated regulatory (Treg) and effector (Teff) T cells were harvested after three days. Experiments were performed with three parallel samples from each condition following the manufacturer's protocol. Slightly higher levels of sIL-1R2 in supernatants of psoriatic T cells were detected compared to healthy samples; however, no significant difference was observed.

After determining the cell surface decoy IL-1 receptor expression, we examined whether Treg and Teff cells from control and psoriatic individuals released soluble IL-1R2 into the supernatant upon activation. The concentration of IL-1R2 in the supernatant of the cells was determined by ELISA in resting state and after three days of CD3/CD28 activation. We detected a notable release of IL-1R2 protein, especially following activation. Although Teff cells secreted higher amounts of the protein into the supernatant than Treg cells, the differences were not statistically significant either in healthy or in psoriatic cells (Figure 4). Psoriatic T cells produced slightly but not significantly higher levels of the sIL-1R2 protein compared to healthy counterparts.

## 4. Discussion

Psoriasis is an inflammatory skin disease, with complex dysregulation of cutaneous immunity [17]. Psoriatic plaque formation is initiated by components of the skin innate immune system and sustained by the abnormal interaction of skin resident cells with cells of the hematopoietic system [1, 18]. T cells have been implicated as key players in the maintenance of psoriasis and the pathogenesis appears to involve a cytokine network centered on IL-17/IL-23 and TNF*α* [19–22].

Here we provide evidence that members of the interleukin-1 receptor family are differentially expressed in psoriatic and healthy peripheral blood T cells. While the proportions of regulatory, memory, naïve, and naïve regulatory T cell populations within the CD4^+^ T cell pool are similar in psoriatic and healthy individuals in the peripheral blood; differences in the expression of the IL-1 receptors, becoming prominent especially upon activation, may contribute to the pathogenesis of psoriasis. Here we also provide additional information on effector/regulatory T cell characteristics by showing that there are fundamental differences between the expression of the signal transmitting and decoy IL-1 receptors on Treg and Teff cells.

Interleukin-1 is one of the cytokines linking innate and adaptive immune responses, and it has long been implicated in the pathogenesis of psoriasis [10, 23], with recent findings further supporting this notion [24]. IL-1*α* activity within psoriatic lesions has been shown to be reduced, relative to both normal epidermal levels [23] and nonlesional skin [10, 24]. On the other hand, increased IL-1*β* production has been reported in psoriatic lesional skin [24]. A highly inducible IL-1R2 receptor expression was observed in epidermal cells, being overexpressed in psoriatic lesions relative to healthy control biopsies [25]. More recently, increased serum levels of the IL-1 receptor antagonist were detected in patients with psoriasis compared to matched controls [26]. Expression of several novel members of the IL-1 family (IL-1F6, IL-1F8, IL-1F9, and IL-1F5) was increased 2 to 3 orders of magnitude in psoriasis plaque versus uninvolved psoriatic skin, inducing the expression of antimicrobial peptides and matrix metalloproteinases in epidermal cells [27]. Chemical irritation of murine skin overexpressing the IL-1 family member IL-1F6 leads to an inflammatory condition similar to human psoriasis [28]. Clearly, from these studies, the significant role for members of the IL-1 family in psoriasis can be established. Differential expression of several ligands and receptors of the IL-1 family have extensively been investigated in psoriasis; however, the possible interaction of immune cells with cells of the epidermis via these cytokines/cytokine receptors has not yet been looked at.

IL-1*α* and IL-1*β* both bind and activate the same receptor [29], stimulating the release of several other proinflammatory cytokines, such as TNF*α* and IL-6, and inducing a Th17 bias in the cellular adaptive responses [13]. Both TNF*α* and the IL-23/Th17 axis are strongly implicated in the pathogenesis of psoriasis [30, 31], while IL-6 has recently been shown to be important in preventing immune suppression by regulatory T cells [14]. Thus, several lines of evidence indicate that IL-1 may directly and indirectly contribute to inflammatory processes in psoriasis.

Apart from the signal transmitting receptor (IL-1R1), IL-1*α* and IL-1*β* can bind to several other members of the IL-1 receptor family. IL-1 receptors have recently been detected on *in vitro* expanded human Treg cells [32]. IL-1R1 is a signalling receptor for IL-1, while IL-1R2 neutralises IL-1 either as a surface decoy receptor or as a cleaved and secreted receptor isoform [9]. IL-1R1 is continuously expressed on resting Treg cells, whereas upon activation it is upregulated on other T cell subpopulations as well, while maintaining preferential expression on the Treg subset [15]. Our results are in concordance with recent findings about Tregs neutralising IL-1*β* activity; thus it is likely that preferential expression of IL-1R2 by these cells may possibly contribute to their suppressive functions.

## 5. Conclusions

In our study, T cells stimulated through the TCR displayed IL-1R1 expression profiles similar to previously published results; that is, although Treg cells maintain the highest levels, all CD4^+^ T cell subsets upregulate their IL-1R1 expression. Interestingly, while under resting conditions the difference between IL-1R1 expressing healthy and psoriatic T cells is minimal, it becomes significantly higher in activated psoriatic cells compared to activated healthy controls. In light of recent data that IL-1*β*, in combination with IL-2, can convert natural human Treg cells into Th17 lineage cells [33], it is tempting to speculate that psoriatic T cells in an IL-1-rich environment, such as the inflamed skin, may be more likely to transform to effector cells than Treg cells of nonpsoriatic individuals. Collectively, our findings suggest that the differential expression of IL-1 receptors on psoriatic T cells may contribute to psoriasis development.
